# Developing and validating a Modified Cachexia Index to predict the outcomes for colorectal cancer after radical surgery

**DOI:** 10.1038/s41430-024-01469-x

**Published:** 2024-07-10

**Authors:** Qinggang Yuan, Lixiang Liu, Kai Wang, Shizhen Zhou, Ji Miao, Bo Gao, Chao Ding, Wenxian Guan

**Affiliations:** 1 Department of General Surgery, Nanjing Drum Tower Clinical College of Xuzhou Medical University, Nanjing, Jiangsu China; 2 Department of General Surgery, Nanjing Drum Tower Hospital, Affiliated Hospital of Medical School, Nanjing University, Nanjing, Jiangsu China; 3 Nanjing Drum Tower Hospital Clinical College of Nanjing University of Chinese Medicine, Nanjing, Jiangsu China; 4 Department of Clinical Nutrition, Nanjing Drum Tower Hospital, Affiliated Hospital of Medical School, Nanjing University, Nanjing, Jiangsu China

**Keywords:** Colorectal cancer, Risk factors, Colorectal cancer, Biomarkers

## Abstract

**Background:**

It was reported that the cachexia index (CXI: $\frac{{\rm{ALB}}* {\rm{SMI}}}{{\rm{NLR}}}$) was an essential index for predicting the prognosis of tumor patients. However, since for SMI needs to be measured by CT imaging methods and its calculation was inconvenient. Thus, we developed a modified cachexia index (mCXI: $\frac{{\rm{ALB}}}{{\rm{NLR}}* {\rm{UCR}}}$). The purpose of this study was to evaluate the association between mCXI and prognosis in patients with colorectal cancer.

**Methods:**

An analysis of 215 patients with newly diagnosed colorectal cancer was carried out retrospectively. An optimal cut-off value of mCXI was established by the receiver operating characteristic (ROC) curves for predicting prognosis. Prognostic implications of mCXI were investigated using Kaplan–Meier curves and Cox regression analysis. A comparative assessment of the predictive capacity between mCXI and the CXI was performed using time-dependent receiver operating characteristic analysis.

**Results:**

Patients were classified into two groups based on the cut-off value of mCXI: the LOW mCXI group (*n* = 60) and the HIGH mCXI group (*n* = 155). The 3-year Overall survival (OS) (76.6% vs 96.7%, *p* < 0.01) and 3-year Recurrence-free survival (RFS) (68.3% vs 94.1%, *p* < 0.01) were significantly worse in the LOW mCXI group in contrast to that in the HIGH mCXI group. In Cox multivariate regression analysis, mCXI was an independent prognostic factor for OS (HR = 8.951, 95%CI: 3.105–25.807, <0.01). Moreover, compared with CXI (AUC = 0.723), mCXI (AUC = 0.801) has better predictive efficacy, indicating that mCXI is more suitable for prognostic assessment.

**Conclusions:**

The mCXI significantly correlated with survival outcomes for colorectal cancer patients after radical surgery.

## Introduction

Colorectal cancer ranks the third most prevalent cancer worldwide and is the second leading cause of cancer-related mortality [1]. The incidence of cancer cachexia affected approximately 50% of individuals diagnosed with colorectal cancer [2], with 20% of cancer-related deaths attributed to cachexia [3]. Moreover, cancer cachexia acts as a prognostic marker for patients with colorectal cancer [1].

Cancer cachexia is a multifactorial syndrome characterized primarily by a persistent deficit of skeletal muscle [2]. Patients with cancer cachexia often experience progressive decline in physical functioning [3], reduced tolerance towards anticancer therapy [4], systemic inflammatory response [5] and negative protein-energy balance [6]. Despite its clinical significance, diagnostic criteria for cancer cachexia was still not in uniform and was mainly related to factors such as weight changes, CRP level and muscle level [7]. In recent years, cachexia index(CXI) has been developed, which was derived from the calculation of skeletal muscle index (SMI) multiplied by serum albumin divided by the neutrophil-lymphocyte ratio(NLR) [8]. At present, the calculation of SMI requires measuring of skeletal muscle area at the third lumbar vertebra level on an abdominal CT scan, while the NLR represents the quotient of serum neutrophil count to serum lymphocyte count. These clinical indicators not only showed independent associations with outcomes in colorectal cancer patients [9–11], CXI is actually the product of the combination of skeletal muscle, nutritional status and body systemic inflammatory status, and could help reflect the comprehensive status involving the above status to some extent. The CXI has been proven as a useful tool in the evaluation of cachexia and is significantly related to survival in patients with cancer such as hepatocellular carcinoma [12], gastric cancer [13], colorectal cancer [14], and diffuse large B-cell lymphoma [8].

Although SMI assessment was primarily based on the results of CT scans, the measurement procedure is time-consuming and laborious, even though patients with colorectal cancer routinely undergo preoperative abdominal CT scans. To overcome these limitations of CT scans in body composition assessment, we tried to identify serum metabolic indexes as potential alternatives. In particular, we focused on the Urea–Creatinine Ratio (UCR), which had a significant negative correlation with SMI as reported [15]. Blood creatinine, primarily stored in muscle tissue, declines in paralleled with muscle catabolism [16]. Blood creatinine can serve as an indicator of the body’s skeletal muscle content, yet its accuracy is interfered by renal function, thereby restricting its utility as a reliable biomarker for assessing skeletal muscle metabolism [17]. Urea-corrected creatinine, briefly as UCR, is obtained by calculating the serum urea to creatinine ratio, offering a method to estimate skeletal muscle mass. The study conducted by Haines and Gao et al. [15, 17] indicated that UCR shows lower sensitivity to factors unrelated to muscle atrophy, making it more suitable for reflecting skeletal muscle level Additionally, Tufan et al. [18] noted that UCR can be used to assess malnutrition. This may be related to the fact that elevated serum urea reflects, in part, a shift in the body’s metabolism towards the hydrolysis of muscle proteins. In a study of intensive care unit patients by Haynes et al., CT scans of 107 patients showed an elevated UCR consistent with a progressive decrease in muscle mass [15]. Therefore, we derived a novel evaluation index, mCXI, which was calculated as a ratio of serum albumin to NLR and UCR. Similar to CXI, the mCXI comprehensively assess malignancy in multiple aspects, and its simplicity facilitates routine repetition of clinical evaluation. However, the prognostic value of mCXI in colorectal cancer patients is unclear. Therefore, this study aimed to investigate the correlation between mCXI and survival outcomes among patients after radical surgery for colorectal cancer, as well as to compare its accuracy in predicting prognosis with that of CXI.

## Materials and methods

### Patients

Data from patients newly diagnosed with colorectal cancer who underwent radical surgical treatment between January 2017 and January 2019 at either Nanjing Drum Tower Hospital or General Hospital of Eastern Theater Command were analyzed retrospectively. Inclusion criteria: (1) Pathologically confirmed diagnosis of colorectal cancer; (2) Radical surgery conducted at the hospital; (3) Preoperative CT scans of the abdomen carried out at our hospital. Exclusion criteria:(1) Patients who the postoperative pathological stage was four stages; (2) Patients received neoadjuvant therapy before surgery;(3) Patients with incomplete medical records. Finally, the study included 215 patients. The Clinical Research Ethics Committee of Nanjing Drum Tower Hospital or General Hospital of Eastern Theater Command approved this observational study.

Upon admission to the hospital, patients undergo a standard and rigorous clinical process. The process includes routine examination, preoperative communication with the patient, signing the consent form for surgery and the consent form for clinical sample collection (consent to use the patient’s case data for relevant analyses and research). In this process, no additional tests and costs will be added to the patient, we protect the patient’s privacy, rights and interests, and the patient voluntarily chooses to agree or refuse.

### Calculation of CXI and mCXI

The CXI was determined using the following procedure: $\frac{{\rm{ALB}}* {\rm{SMI}}}{{\rm{NLR}}}$ [13]. The mCXI was calculated by adopting the following formula: $\frac{{\rm{ALB}}}{{\rm{NLR}}* {\rm{UCR}}}$. The neutrophil count to lymphocyte count ratio was used to calculate the NLR. The UCR was calculated as the ratio of urea nitrogen to creatinine, all derived from blood samples collected within the seven days before surgery (Table 1).

**Table 1:** Clinicopathological characteristics of 215 patients wi1th colorectal cancer after surgery.

Variable	ALL (215)	Low mCXI (60)	High mCXI (155)	*P* value
Age (years)	58.4 ± 12.8	60.0 ± 14.4	57.8 ± 12.1	0.268
Male sex, *n* (%)	124 (57.7%)	29 (48.3%)	95 (61.2%)	0.085
BMI (kg/m2)	22.9 ± 3.2	22.1 ± 3.5	23.2 ± 3.1	0.02
SMI (cm2/m2)	43.1 ± 4.3	40.6 ± 4.7	44.1 ± 3.7	<0.01
WBC (×10^9^/L)	5.8 ± 1.9	6.1 ± 2.0	5.7 ± 1.8	0.191
Neutrophil (×10^9^/L)	3.7 ± 1.8	4.6 ± 2.5	3.4 ± 1.3	<0.01
Lymphocyte (×10^9^/L)	1.6 ± 0.6	1.2 ± 0.6	1.7 ± 0.6	<0.01
Platelet (×10^9^/L)	216.9 ± 83.7	213.0 ± 101.0	218.4 ± 77.8	0.674
Hemoglobin (g/L)	122.7 ± 24.6	117.0 ± 24.3	124.9 ± 24.4	0.035
Total cholesterol	4.3 ± 1.1	4.2 ± 1.2	4.4 ± 1.0	0.182
Albumin (g/L)	41.3 ± 4.3	39.8 ± 5.4	41.9 ± 3.7	0.007
Creatinine	65.5 ± 18.0	59.9 ± 18.9	67.7 ± 17.1	0.004
Blood urea nitrogen	5.2 ± 1.5	5.7 ± 2.2	5.0 ± 1.2	0.021
CRP (mg/L)	8.6 ± 19.8	13.1 ± 26.1	6.9 ± 16.5	0.085
CEA (ng/ml)	5.9 ± 10.1	5.2 ± 5.7	6.2 ± 11.4	0.493
CA199 (U/ml)	24.8 ± 54.6	18.7 ± 26.2	27.2 ± 62.2	0.305
Tumor site, *n* (%)				0.350
Colon	122 (56.7%)	31 (51.7%)	91 (58.7%)	
Rectum	93 (43.3%)	29 (48.3%)	64 (41.3%)	
Comorbidities, yes, *n* (%)	69 (32.1%)	17 (28.3%)	52 (33.5%)	0.463
Postoperative complications, yes, *n* (%)	34 (15.8%)	9 (15.0%)	25 (16.1%)	0.839
Surgical method				0.874
Open	49 (22.8%)	16 (26.7%)	43 (27.7%)	
Laparoscopy	156 (77.2%)	44 (73.3%)	112 (72.3%)	
Tumor size (cm)	4.1 (1.8 ± 0.1)	4.3 (2.0 ± 0.3)	4.086 (1.8 ± 0.1)	0.617
Depth of invasion				0.835
T1–2	45 (20.9%)	12 (20%)	33 (21.3%)	
T3–4	170 (79.1%)	48 (80%)	122 (78.7%)	
CRC stage, *n* (%)				0.907
I	36 (16.7%)	9 (15.0%)	27 (17.4%)	
II	89 (41.4%)	25 (41.7%)	64 (41.3%)	
III	90 (41.9%)	26 (43.3%)	64 (41.3%)	

Group differences were compared using the χ2 test or ANOVA.

*mCXI* modified cachexia index, *mCXI* modified cachexia index, *BMI* body mass index, *SMI* Skeletal muscle mass index, *WBC* white blood cell, *CRP* C-reactive protein, *CEA* carcinoembryonic antigen, *CA199* Carbohydrate antigen199, *NLR* neutrophil-to-lymphocyte ratio, *CXI* cachexia index.

### Follow up

Patients who underwent surgery for colorectal cancer and were discharged from the hospital were subjected to regular follow-up via telephone at three-month intervals. Follow-up endpoints were the occurrence of death, recurrence, and patient status at the 3-year cutoff time point. Regular hospital appointments and follow-up were necessary. The outcome was mortality from any cause of illness and the medically confirmed recurrence.

### Statistical analysis

Statistical analyses were performed using IBM SPSS Statistics for Windows, Version 26.0(Armonk, NY: IBM Corp). When appropriate, Pearson’s or Spearman’s correlation coefficients were utilized, to examine the correlations between parameters. Group differences were compared using the χ2 test or ANOVA. The Kaplan–Meier curve investigated the 3-year OS (Overall survival) and 3-year RFS (Recurrence-free survival). Univariate Cox proportional hazard models were also utilized to explore the OS. Then, variables with *P* values < 0.05 during and univariate analysis undergo further analysis in multivariate analysis. The efficacy of prognosis prediction was evaluated using a time-dependent receiver operating characteristic (ROC) curve. Cut-offs were calculated with the Youden index. Statistical significance was set at *P* < 0.05. We first built a Cox proportional hazards model and examined the survival curves, Schoenfeld residuals, and log–log plots to detect any violations in proportionality assumption, and the degrees of freedom needed for the restricted cubic spline function used for the baseline hazard rate and for the potential time-dependent effects. The final model was chosen using the AIC [19]. In the analysis of the Kaplan–Meier curves and Cox regression models, we used the right-censoring treatment of patient withdrawals that is common in survival analyses.

## Results

### Clinical characteristics

The baseline characteristics of the two groups stratified by mCXI are presented in Table 1. Overall, 215 patients diagnosed with colorectal cancer were involved in this research, comprising 124 (57.7%) males and 91 (42.3%) females. The participants’ mean age was 58.4 years (±12.8 years). Utilizing time-dependent ROC curves, patients with mCXI <161.4 were categorized into the low mCXI group, while those with mCXI ≥161.4 were classified into the high mCXI group. Compared with patients who exhibit high mCXI, patients in the low mCXI group had lower levels of BMI (low mCXI vs high mCXI: 22.1 ± 3.5 kg/m^2^ vs 23.2 ± 3.1 kg/m^2^, *p* = 0.02), SMI (low mCXI vs high mCXI: 40.6 ± 4.7 cm^2^/m^2^ vs 44.1 ± 3.7 cm^2^/m^2^, *p* < 0.01), hemoglobin (low mCXI vs high mCXI: 117.0 ± 24.3 g/L vs 124.9 ± 24.4 g/L, *p* < 0.01), lymphocytes (low mCXI vs high mCXI: 1.2 ± 0.6 × 109/L vs 1.7 ± 0.6 × 109/L, *p* < 0.01), more elevated neutrophils (low mCXI vs high mCXI: 4.6 ± 2.5 × 109/L vs 3.4 ± 1.3 × 109/L, *p* < 0.01), Albumin(g/L) (low mCXI vs high mCXI: 39.8 ± 5.4 vs 41.9 ± 3.7, *p* < 0.01), Creatinine(low mCXI vs high mCXI: 59.9 ± 18.9 umol/L vs 67.7 ± 17.1 umol/L, *p* < 0.01), Blood urea nitrogen(low mCXI vs high mCXI: 5.7 ± 2.2 mmol/L vs 5.0 ± 1.2 mmol/L, *p* < 0.01) (Table 1). There was no significant difference in age, gender, white blood cell count, platelets, CRP, CEA, CA199, tumor location, postoperative pathological stage, presence of underlying diseases, and postoperative complications between the two groups.

### mCXI and survival

**Fig. 1:**
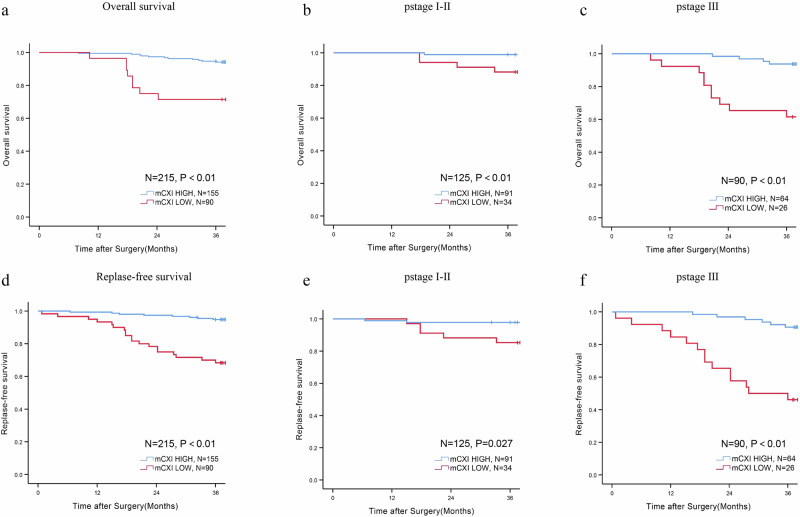
Prognosis of different mCXI subgroups. Kaplan–Meier curves for overall survival and replase-free survival in HIGH mCXI or LOW mCXI groups (**a**, **d**), pStageI-II patients (**b**, **e**), and pStageIII patients (**c**, **f**).

With a median follow-up period of 45 months (range 8–85 months), the 3-year OS (76.6% vs 96.7%, *p* < 0.01) and 3-year RFS (68.3% vs 94.1%, *p* < 0.01) in the mCXI low group were considerably lower than those in the mCXI high group (Fig. 1). We further subgroup analyzed the effect of mCXI on OS and RFS in patients with different stages. Notably, patients with CRC stages 1–2 and 3 in the low mCXI group had significantly poorer OS and RFS than those in the high mCXI group (Fig. 1).

### Cox regression analysis

To examine the impact of various clinical factors on patients’ OS and RFS, we performed univariate Cox regression analysis. We discovered that BMI ≥ 24 (HR = 0.199, 95% CI: 0.046–0.860, *P* = 0.031) and hemoglobin ≥90 (HR = 0.354, 95% CI: 0.127–0.982, *P* = 0.046) were more beneficial for 3-year OS, whereas CRC stage III (HR = 4.100, 95% CI: 1.477–11.385, *P* = 0.007) and mCXI Low (HR = 8.179, 95% CI: 2.945–22.719 *P* < 0.01) exerted more risk on 3-year OS (Table 2). High BMI (HR = 0.365, 95% CI: 0.139–0.959, *P* = 0.041), high CEA level (HR = 2.279, 95% CI: 1.031–5.039, *P* = 0.042), CRC III of tumor severity (HR = 3.845, 95% CI: 1.690–8.747, *P* < 0.01), and low mCXI (HR = 6.399, 95% CI: 2.893–14.154, *P* < 0.01) were significantly associated with 3-year RFS (Table 3). After adjusting statistically significant variables in the multivariate analysis, preoperative hemoglobin (HR = 0.300, 95% CI: 0.104–0.869, *P* = 0.027), patient’s CRC stage (HR = 4.402, 95% CI: 1.544–12.557, *P* = 0.006) and mCXI (HR = 8.951, 95% CI: 3.105–25.807, *P* = < 0.01) were highly correlated with OS (Table 2). Preoperative CEA level (HR = 2.382, 95% CI: 1.071–5.300, *P* = 0.033), patient tumor stage (HR = 4.001, 95% CI: 1.721–9.302, *P* < 0.01), and mCXI (HR = 6.767, 95% CI: 3.017–15.176, *P* < 0.01) were significantly correlated with the 3-RFS were correlated considerably (Table 3).

**Table 2:** Uni- and multivariate analyses of factors associated with 3-year overall survival (OS) in patients with colorectal cancer.

		Univariate analysis			Multivariate analysis		
Variable		HR	95% CI	*P* value	HR	95%CI	*P* value
Age (years)	≥65/<65	1.829	0.743–4.501	0.189			
Sex	Female/Male	1.551	0.630–3.817	0.339			
BMI (kg/m2)	≥24/<24	0.199	0.046–0.860	0.031	0.361	0.081–1.598	0.179
Tumor site	Colon/Rectum	1.051	0.423–2.613	0.915			
Comorbidities	Yes/NO	2.594	0.756–8.904	0.130			
WBC (×10^9^/L)	≥10/<10	1.375	0.184–10.301	0.757			
Lymphocyte(×10^9^/L)	≥3.5/<3.5	0.266	0.036–1.993	0.197			
Neutrophil (×10^9^/L)	≥1.5/<1.5	0.438	0.058–3.279	0.438			
Albumin (g/L)	≥40/<40	0.556	0.226–1.369	0.202			
Platelet (×10^9^/L)	≥300/<300	0.824	0.190–3.568	0.796			
Hemoglobin (g/L)	≥90/<90	0.354	0.127–0.982	0.046	0.300	0.104–0.869	0.027
CRP (mg/L)	>10/≤10	1.608	1.608–6.959	0.525			
CEA (ng/ml)	≥3.4/<3.4	2.366	0.899–6.225	0.081			
CA199 (U/ml)	≥37/<37	1.817	0.243–13.613	0.561			
Postoperative complications	Yes/NO	1.017	0.296–3.489	0.979			
TNM stage	III/I,II	4.100	1.477–11.385	0.007	4.402	1.544–12.557	0.006
mCXI	Low/High	8.179	2.945–22.719	<0.01	8.951	3.105–25.807	<0.01

*BMI* body mass index, *WBC* white blood cell, *CEA* carcinoembryonic antigen, *CA199* Carbohydrate antigen199, *HB* hemoglobin, *CRP* C-reactive protein, *CXI* cachexia index, *mCXI* modified cachexia index.

**Table 3:** Uni- and multivariate analyses of factors associated with replase-free survival (RFS) rates in patients with colorectal cancer.

		Univariate analysis			Multivariate analysis		
Variable		HR	95% CI	*P* value	HR	95%CI	*P* value
Age (years)	≥65/<65	1.018	0.988–1.049	0.250			
Sex	Female/Male	0.739	0.054–1.151	0.222			
BMI (kg/m2)	≥24/<24	0.365	0.139–0.959	0.041	0.515	0.190–1.395	0.192
Tumor site	Colon/Rectum	0.930	0.438–1.979	0.852			
Comorbidities	Yes/NO	1.484	0.633–3.477	0.364			
WBC (×10^9^/L)	≥10/<10	2.019	0.478–8.523	0.339			
Lymphocyte(×10^9^/L)	≥3.5/<3.5	0.393	0.053–2.893	0.350			
Neutrophil (×10^9^/L)	≥1.5/<1.5	0.645	0.088–4.753	0.667			
Albumin (g/L)	≥40/<40	0.740	0.345–1.588	0.440			
Platelet (×10^9^/L)	≥300/<300	1.206	0.364–3.994	0.760			
Hemoglobin (g/L)	≥90/<90	0.570	0.216–1.506	0.257			
CRP (mg/L)	>10/≤10	1.521	0.458–5.052	0.493			
CEA (ng/ml)	≥3.4/<3.4	2.279	1.031–5.039	0.042	2.382	1.071–5.300	0.033
CA199 (U/ml)	≥37/<37	1.367	0.324–5.768	0.670			
Postoperative complications	Yes/NO	1.051	0.618–1.787	0.854			
TNM stage	III/I,II	3.845	1.690–8.747	<0.01	4.001	1.721–9.302	<0.01
mCXI	Low/High	6.399	2.893–14.154	<0.01	6.767	3.017–15.176	<0.01

*BMI* body mass index, *WBC* white blood cell, *CEA* carcinoembryonic antigen, *CA199* Carbohydrate antigen199, *HB* hemoglobin, *CRP* C-reactive protein, *CXI* cachexia index, *mCXI* modified cachexia index.

### Prediction accuracy comparison between mCXI and CXI

**Fig. 2:**
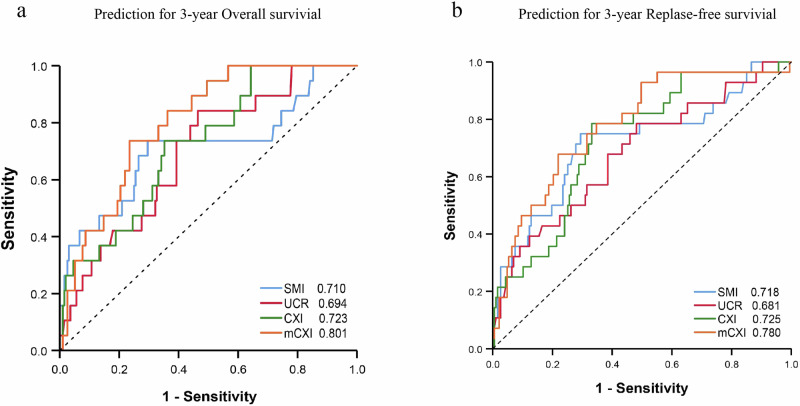
ROC curves for different indicators. The time-dependent curves of SMI, 1/UCR, CXI and mCXI for predicting 3-year overall survival (**a**) and 3-year replase-free survival (**b**).

Using time-dependent ROCs to predict 3-year OS in patients as a whole. The areas under the curves (AUC) for SMI, 1/UCR, CXI and mCXI were 0.710 (95% CI 0.566–0.855, *P* < 0.01), 0.694 (95% CI 0.579–0.809, *P* < 0.01), 0.723 (95% CI 0.614–0.831, *P* < 0.01) and 0.801 (95% CI 0.717–0.885, *P* < 0.01) respectively (Fig. 2). Furthermore, when predicting the 3-year RFS, the AUC was 0.718 (95% CI 0.605–0.831, *P* < 0.01), 0.681 (95% CI 0.571–0.792, *P* < 0.01), 0.725 (95% CI 0.631–0.820, *P* < 0.01), 0.780 (95% CI 0.689–0.871, *P* < 0.01) (Fig. 2). After Delong test, we found that the AUC difference between mCXI and CXI was statistically significant in predicting three-year OS (*P* = 0.01) and three-year RFS (*P* = 0.048). Therefore, in this study, mCXI was the best predictor of survival and recurrence in CRC patients.

## Discussion

Cancer cachexia is a multifactorial syndrome characterized by weight loss, accompanied by the depletion of skeletal muscle and adipose tissue. Significantly, the depletion of skeletal muscle mass is a crucial distinguishing feature of this syndrome [3, 20]. Moreover, malnutrition and systemic inflammation due to tumor progression are vital features associated with cancer cachexia [21]. It is noteworthy that cancer cachexia contributes indirectly to the mortality of 20% of cancer patients, with its incidence reported to be approximately 50% in colorectal cancer patients [22]. Colorectal cancer patient’s prognosis and quality of life are substantially affected by cancer cachexia [23].

The CXI, regarded as a potential biomarker of cancer cachexia [8], was derived from the formula SMI*ALB/NLR [13]. SMI is primarily determined by measuring the skeletal muscle area in the L3 slice level of the abdominal CT [24], serum albumin and NLR dependent on the preoperative blood draw. The assessment of skeletal muscle in the third lumbar spine cross-section on preoperative abdominal CT scan was widely recognized as a reflection of the skeletal muscle mass in the body. Moreover, research has demonstrated the usefulness of SMI as a prognostic factor for patients with colorectal cancer [25–27]. Moreover, decreased albumin levels may indicate elevated inflammation in patients with tumors [28]. Consequently, using albumin levels to predict the prognosis of tumor patients is also well-documented in scientific literature [29, 30]. The NLR, an inflammatory indicator reflecting systemic inflammation in the body, has proven highly valuable in predicting prognosis of patients with colorectal cancer [31, 32]. Therefore, CXI effectively assesses the degree of cancer cachexia in terms of skeletal muscle status, nutritional status, and systemic inflammation level, thereby enabling the prediction of patient prognosis. As the CXI necessitates preoperative abdominal CT and blood test reports, it remains applicable to all preoperative colorectal cancer patients. However, the sophisticated operation in muscle mass measurement at the L3 section of abdominal CT and the tremend workload exerted on radiologists largely restricted its use in the clinic. In addition, postoperative CT scan in routine follow-up period was often unnecessary, leading to the inabitity to reassess SMI after surgery. To address this issue, we employed the serum biomarker index UCR, which demonstrated a significant negative correlation with SMI, as a replacement for SMI in the malignancy index.

UCR was selected as a substitute for SMI in assessing malignant disease for two main reasons. Firstly, UCR offers a computationally simpler option for acquiring SMI [33]. Secondly, UCR functions as an indicator of catabolism, not only providing a more comprehensive representation of whole-body skeletal muscle content compared to SMI [30] but also capturing the ongoing muscle breakdown in the body [15]. Moreover, the prognostic efficacy of mCXI was observed to be excellent to that of CXI (Fig. 2). Therefore, the mCXI has superior advantages, such as more easier, more accurate and timely prognostic potential than CXI.

This study has several limitations. Firstly, Our study used a retrospective design to collect data from past records, and patients selected for inclusion in the analysis may have been affected by factors not controlled for in the study, and thus may have suffered from selection bias. Secondly, Our study relied on data from two specific hospitals, involved a homogenous ethnic region, and had a limited sample size, which may not be representative of the wider population and lack diversity. Thirdly, In addition, our study included only stage III patients and excluded stage IV, which resulted in a partial selection bias. Therefore, further prospective studies are warranted to validate the predictive importance of the mCXI.

## Conclusion

In summary, the mCXI showed a significant association with the postoperative prognosis of patients with colorectal cancer. Moreover, in the context of predicting the prognosis following radical surgery for colorectal cancer, the mCXI demonstrated outstanding predictive ability to that of the CXI, signifying its effectiveness as a valuable prognostic indicatiors in the clinic.

## Data Availability

The patient data utilized in this study is available in the Excel spreadsheet.
